# Opportunistic osteoporosis screening in primary hyperparathyroidism using routine CT: validation of a standardized vertebral cBMD index

**DOI:** 10.3389/fendo.2026.1710379

**Published:** 2026-02-13

**Authors:** Jiayi Pu, Miao Wei, Wenqin Zhou, Wen Li, Yan Xiao, Jia Xie, Bangyuan Long, Fajin Lv

**Affiliations:** 1 Department of Radiology, The First Affiliated Hospital of Chongqing Medical University, Chonqing, China; 2 Department of Radiology, Yubei District Hospital of Traditional Chinese Medicine, Chonqing, China; 3 Department of Radiology, Bishan Hospital of Chongqing Medical University, Chonqing, China; 4 Department of Radiology, Beibei District Hospital of Traditional Chinese Medicine, Chonqing, China; 5 Department of Radiology, Chongqing General Hospital, Chongqing, China

**Keywords:** bone mineral density, opportunistic CT, osteoporosis, percentage change in bone mineral density (cBMD), primary hyperparathyroidism, quantitative CT

## Abstract

**Purpose:**

To evaluate the diagnostic performance of a routine CT-derived vertebral bone density index, the standardized percentage change in bone mineral density (cBMD), for detecting osteoporosis in patients with primary hyperparathyroidism (PHPT), and to explore its association with parathyroid hormone (PTH) compared to Quantitative CT (QCT).

**Materials and methods:**

This retrospective study included 175 consecutive patients with biochemically confirmed PHPT. QCT–derived volumetric bone mineral density (vBMD), obtained using asynchronous calibration, served as the reference standard. cBMD was calculated from routine non-contrast CT images using a phantom-less normalization method. Diagnostic performance was evaluated using receiver operating characteristic (ROC) analysis. Multivariable logistic regression was used to assess the incremental diagnostic value of cBMD beyond demographic and biochemical variables, and multivariable linear regression was performed to compare independent determinants of cBMD and QCT-vBMD.

**Results:**

Vertebral cBMD showed a strong linear correlation with QCT-vBMD across T12–L3 (r > 0.95, P< 0.001). For osteoporosis detection, L2-cBMD achieved an area under the ROC curve (AUC) of 0.992, with 100.0% sensitivity and 92.5% specificity. Incorporation of L2-cBMD into multivariable models significantly improved diagnostic performance compared with models including demographic and biochemical variables alone (AUC increase from 0.937 to 0.996, P< 0.001). In linear regression analyses adjusted for age and body mass index, cBMD showed an independent association with serum PTH levels (P = 0.010), whereas QCT-vBMD did not (P = 0.398).

**Conclusion:**

cBMD is a robust, phantom-less tool derived from routine CT images that demonstrates high diagnostic accuracy for osteoporosis in PHPT. Its association with serum PTH highlights its potential utility as a complementary imaging biomarker for opportunistic bone health screening in this population.

## 1 Introduction

Primary hyperparathyroidism (PHPT) is characterized by autonomous secretion of parathyroid hormone (PTH), resulting in abnormal bone remodeling, progressive bone loss, and an increased risk of fragility fractures (1–4). Skeletal involvement represents a major clinical concern in PHPT and constitutes a key criterion for therapeutic decision-making, including indications for parathyroidectomy (5). Accurate assessment of bone mineral density (BMD) is therefore essential for the clinical management of affected patients.

Dual-energy X-ray absorptiometry (DXA) is currently the most widely used method for BMD assessment in clinical practice (6). However, DXA provides two-dimensional areal BMD measurements and cannot differentiate cortical from trabecular bone compartments. Its results may be influenced by spinal degenerative changes and vascular calcifications, which can limit its accuracy in certain patient populations (7, 8).

Quantitative computed tomography (QCT) enables three-dimensional evaluation of bone and allows direct quantification of trabecular volumetric BMD (vBMD), most commonly at the lumbar spine where trabecular bone is metabolically active (9). Compared with DXA, QCT is less affected by degenerative artifacts and provides compartment-specific information. Accordingly, it is often used in selected clinical scenarios, such as when DXA results are unreliable or when more precise assessment of trabecular bone is required. Nevertheless, conventional QCT relies on dedicated calibration phantoms and specialized software and is associated with higher radiation exposure, which limits its routine use in clinical practice (10).

In recent years, opportunistic assessment of bone health using routine CT scans obtained for other clinical indications has gained increasing attention (11, 12). Building on this concept, we proposed a simplified and phantom-less vertebral bone density index, termed the standardized percentage change in bone mineral density (cBMD). This index can be retrospectively computed from routinely acquired clinical CT images without additional scanning or calibration phantoms by normalizing trabecular attenuation to a fixed reference derived from healthy controls, yielding a unitless percentage deviation. Therefore, the aims of the present study were to (1) validate vertebral cBMD against QCT-derived vBMD in patients with PHPT, and (2) assess its added diagnostic value when incorporated into multivariable models including demographic and biochemical parameters.

## 2 Materials and methods

### 2.1 Study design and participants

This retrospective, single-center study was approved by the institutional ethics committee, with the requirement for informed consent waived. We consecutively included patients clinically diagnosed with PHPT who underwent CT examinations between April 2022 and February 2025 at the First Affiliated Hospital of Chongqing Medical University. At our institution, non-contrast thoracoabdominal CT is routinely performed as part of the clinical workup for PHPT patients, primarily for nephrolithiasis screening and anatomical evaluation, and is not restricted to patients with advanced disease. In addition, QCT-based skeletal assessment is incorporated into routine clinical care in this population, allowing quantitative evaluation of vertebral BMD. Inclusion criteria: (1) confirmed diagnosis of PHPT, including hypercalcemic and normocalcemic phenotypes, based on persistently elevated PTH levels per international guidelines; (2) availability of QCT-derived vertebral vBMD data and corresponding laboratory data; and (3) age ≥18 years. Exclusion criteria: (1) history of other metabolic bone diseases or systemic conditions affecting bone metabolism (e.g. chronic kidney disease or malignancy with bone metastasis); (2) history of lumbar spinal surgery or internal fixation; (3) severe vertebral deformity, compression fracture (Genant grade > 1), or imaging artifacts compromising CT analysis; and (4) prior use of anti-osteoporotic or bone-active medications (e.g. bisphosphonates or glucocorticoids) within the preceding 12 months.

### 2.2 CT Imaging protocol

All examinations were performed on a SOMATOM Force dual-source CT scanner (Siemens Healthineers, Germany) using a thin-slice helical acquisition. Parameters were as follows: detector collimation 192×0.6 mm; tube voltage 120 kVp; automatic tube current modulation; gantry rotation time 0.25 s. Images were reconstructed with a medium soft-tissue convolution kernel (Br40) at 1.0-mm slice thickness and 1.0-mm increment, with a 512×512 matrix and a 32-cm field of view. Sagittal and coronal multiplanar reconstructions (MPRs) were generated from the 1.0-mm data for vertebral alignment and level localization; all cBMD measurements were performed on 1.0-mm axial images. The same acquisition and reconstruction settings were applied to all subjects.

### 2.3 Measurement of bone mineral density

#### 2.3.1 QCT-vBMD measurement

QCT-vBMD of L1–L3 was measured using the Mindways QCT Pro system (Mindways, Austin, TX, USA). Regions of interest (ROIs) were placed semi-automatically within the central trabecular compartment of each vertebral body (**Supplementary Figure S1**). After asynchronous phantom calibration, volumetric BMD was obtained for each level, and the average vBMD across L1–L3 was used as the diagnostic index. Diagnostic thresholds followed internationally accepted criteria (13): osteoporosis, vBMD< 80 mg/cm³; osteopenia, 80 ≤ vBMD< 120 mg/cm³; normal bone mass, vBMD ≥ 120 mg/cm³. For this study, osteoporosis and osteopenia were collectively defined as low bone mass (vBMD< 120 mg/cm³).

#### 2.3.2 cBMD measurement

**Figure 1:**
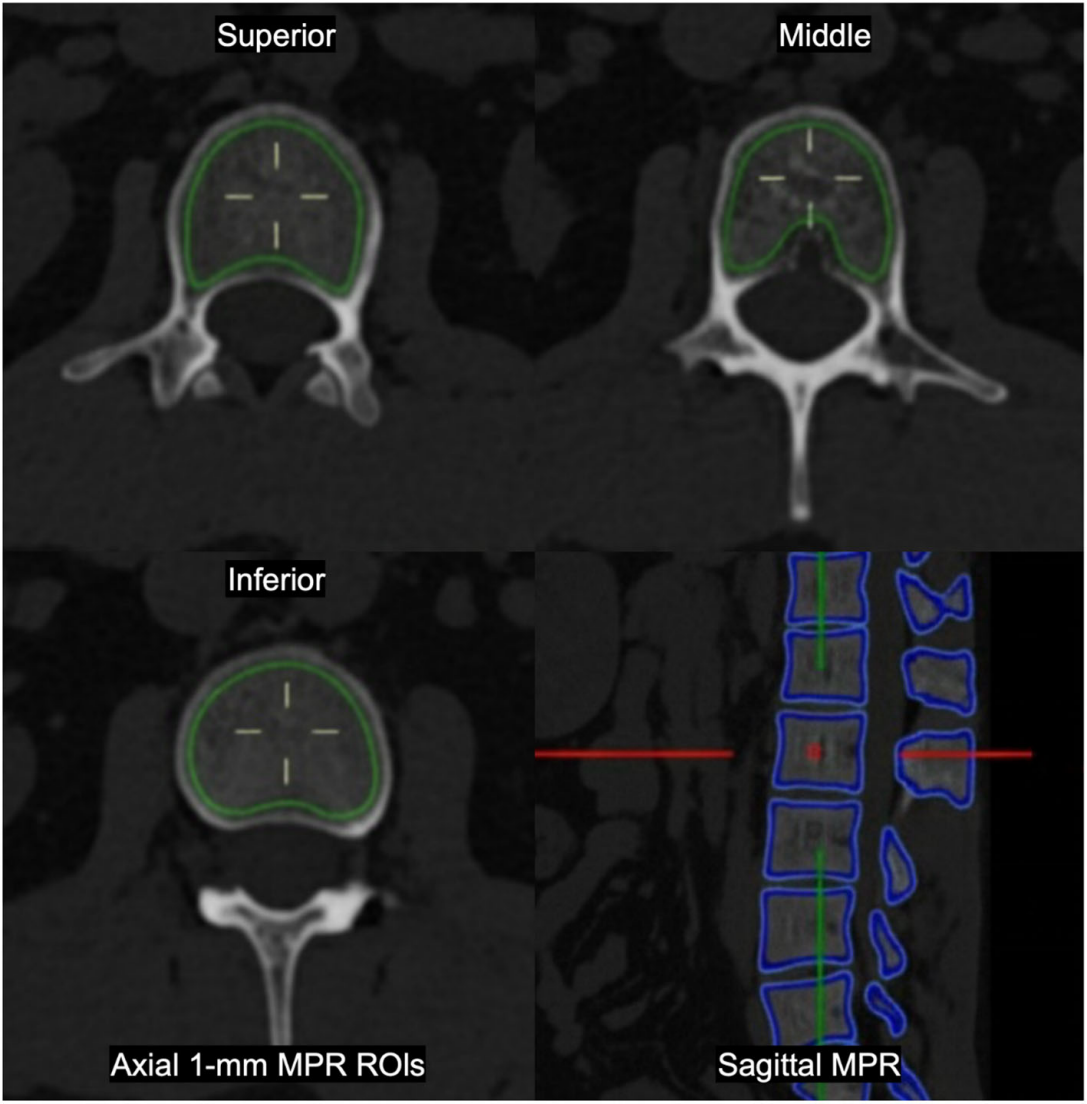
cBMD measurement. Example from one subject (L2 vertebra). Three axial 1-mm slices (superior, middle, and inferior) with manually delineated ROIs encompassing the entire trabecular compartment beneath the cortical shell, excluding cortical bone, bone islands, and the basivertebral venous plexus. CT_mean is the average attenuation of the three slices.


For cBMD measurement, vertebral attenuation was extracted directly from the routine thoracoabdominal protocol described above. For each vertebra from T12 to L3, three axial slices were selected, corresponding to regions adjacent to the superior endplate, the mid-vertebral body, and the inferior endplate. On each slice, a ROI was manually delineated to encompass the entire trabecular compartment beneath the cortical shell, while excluding cortical bone, bone islands, and the basivertebral venous plexus (**Figure 1**). The mean attenuation value across the three slices (CT_mean) was recorded for each vertebra. A reference CT attenuation value (CT_ref, 440 HU) was defined from sagittal multiplanar volume reconstructions (MPVRs) by selecting structurally intact vertebrae from an independent cohort of healthy volunteers. cBMD (unitless, %) was computed as:

$$cBMD=\frac{CT\_mean-\text{CT}\_\text{ref}}{\text{CT}\_\text{ref}}\times 100\%$$

This index represents a relative percentage deviation from the trabecular reference. For exploratory purposes, values were provisionally stratified into five levels, but these thresholds were not externally validated and were excluded from formal analyses.

### 2.4 Statistical analysis

All analyses were performed in R (version 4.3.2). Continuous variables were summarized as mean ± standard deviation (SD). Primary outcomes were osteoporosis and low bone mass defined by QCT, as described above. Correlations between cBMD and QCT-vBMD were assessed using Pearson correlation coefficients, and receiver operating characteristic (ROC) curves were generated to evaluate the diagnostic performance of cBMD for detecting osteoporosis and low bone mass. Stepwise multivariable logistic regression models were constructed to assess the incremental diagnostic value of cBMD for QCT-defined osteoporosis. We selected L2-cBMD *a priori* as the representative vertebral index, given its central anatomical position and lower susceptibility to transitional anatomy or degenerative changes, consistent with common QCT practice. Model 1 included demographic variables (age, sex, body mass index [BMI]); Model 2 additionally incorporated serum calcium, PTH, and 25-hydroxyvitamin D [25(OH)D]; Model 3 further included bone turnover markers (osteocalcin, procollagen type I N-terminal propeptide [PINP], and C-terminal telopeptide of type I collagen [CTX]); and Model 4 added L2-cBMD to evaluate its added diagnostic contribution. Comparisons of the area under the ROC curves (AUCs) between models were conducted using DeLong’s test.

To further compare the associations of cBMD and QCT-vBMD with clinical and biochemical factors, multivariable linear regression analyses were performed. Separate models were constructed with QCT-vBMD and L2-cBMD as dependent variables, respectively. Both models were adjusted for age, sex, BMI, PTH, 25(OH)D, and bone turnover markers (CTX, PINP and BALP). A two-sided P value< 0.05 was considered statistically significant for all analyses.

## 3 Results

### 3.1 Baseline characteristics

A total of 175 patients with PHPT were included and categorized by QCT-vBMD into normal bone mass (n = 88, 50.3%), osteopenia (n = 38, 21.7%), and osteoporosis (n = 49, 28.0%). Baseline demographic and clinical characteristics are summarized in **Table 1**. Age increased stepwise from normal to osteoporosis, indicating an age-related decline in bone mass. The proportion of women was higher in osteopenia and osteoporosis than in the normal group (P = 0.0099). BMI showed no significant group differences.

**Table 1:** Demographic and clinical characteristics of the study cohort stratified by QCT−vBMD status.

Variable	Overall	Normal (≥120 mg/cm³)	Osteopenia (80–120 mg/cm³)	Osteoporosis (<80 mg/cm³)	P value
Sample size, n	175	88	38	49	—
Age, years	50.28 ± 14.54	40.93 ± 10.92	54.82 ± 12.90	63.55 ± 8.07	<0.001
Sex, n (%)					0.010
Male, n (%)	51 (29.1%)	34 (38.6%)	10 (26.3%)	7 (14.3%)	—
Female, n (%)	124 (70.9%)	54 (61.4%)	28 (73.7%)	42 (85.7%)	—
BMI, kg/m²	23.70 ± 3.47	23.64 ± 3.80	23.63 ± 3.12	23.86 ± 3.16	0.930
QCT−vBMD (mg/cm³)	120.18 ± 52.25	163.92 ± 29.61	99.73 ± 12.42	57.50 ± 18.46	<0.001
T12 attenuation, HU	161.42 ± 69.99	217.43 ± 46.98	132.76 ± 19.55	83.05 ± 28.14	<0.001
L1 attenuation, HU	155.35 ± 68.70	211.79 ± 41.95	127.95 ± 17.21	75.24 ± 26.58	<0.001
L2 attenuation, HU	152.81 ± 70.51	210.62 ± 45.25	122.31 ± 16.72	72.64 ± 25.95	<0.001
L3 attenuation, HU	151.89 ± 73.34	211.97 ± 47.57	118.64 ± 19.39	69.76 ± 26.97	<0.001
T12−cBMD, %	-63.31 ± 13.44	-50.58 ± 0.11	-69.83 ± 0.04	-81.13 ± 0.06	<0.001
L1−cBMD, %	-64.69 ± 13.60	-51.87 ± 0.10	-70.92 ± 0.04	-82.90 ± 0.06	<0.001
L2−cBMD, %	-65.27 ± 13.83	-52.13 ± 0.10	-72.20 ± 0.04	-83.49 ± 0.06	<0.001
L3−cBMD, %	-65.49 ± 14.31	-51.83 ± 0.11	-73.04 ± 0.04	-84.15 ± 0.06	<0.001
DXA lumbar T−score	-2.33 ± 1.25	-1.42 ± 0.36	-2.72 ± 1.37	-3.66 ± 0.74	<0.001

Continuous variables are reported as mean ± standard deviation (SD); categorical variables as n (%). P values are from one-way ANOVA (continuous variables) and chi-square test (sex), all two-sided. DXA lumbar T-score was available in a subset only (n = 102).

**Figure 2:**
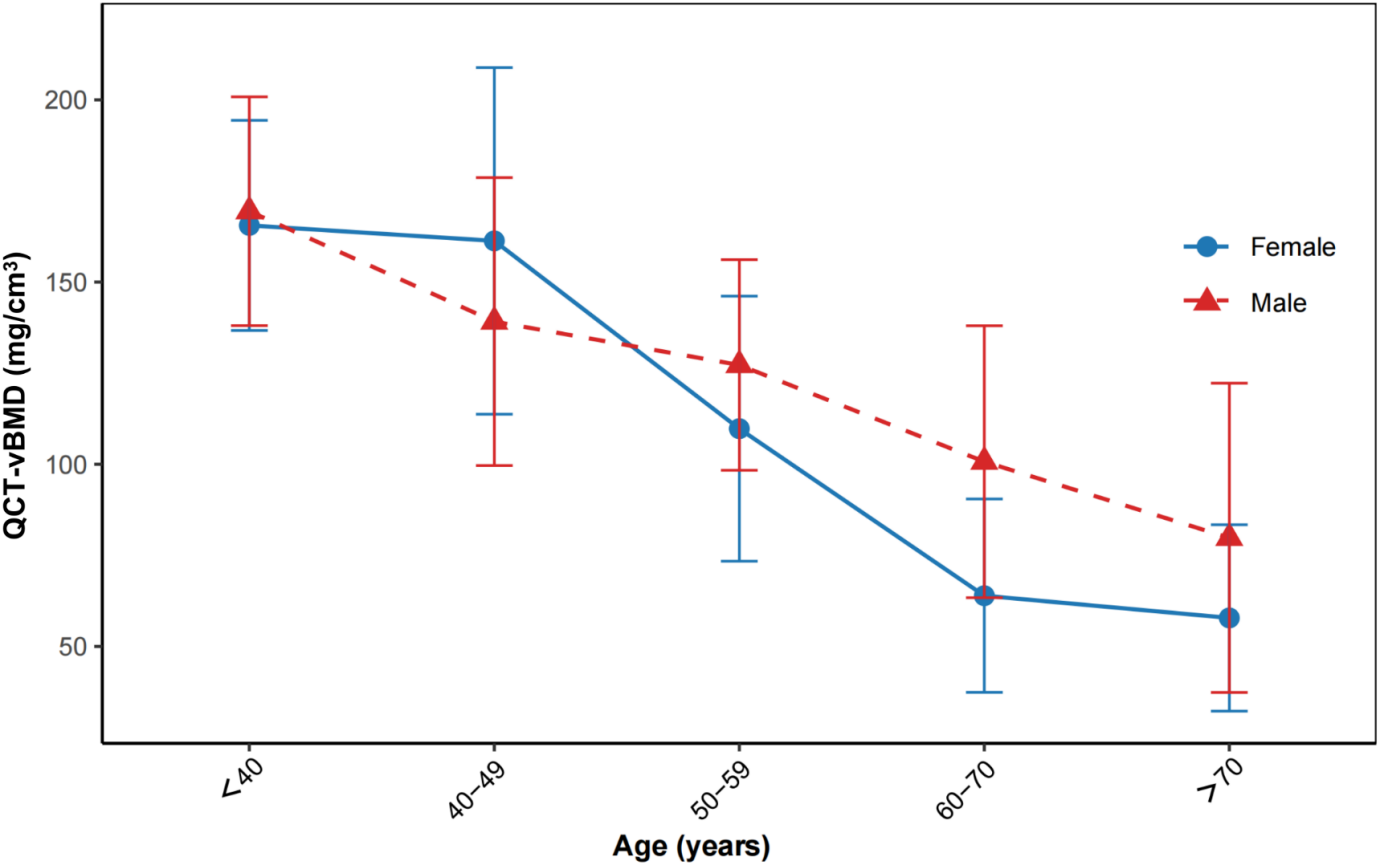
Age- and sex-related variations in lumbar QCT-vBMD in PHPT. Points show group means; error bars indicate 95% confidence intervals. Age bins:<40, 40–49, 50–59, 60–70, >70 years. Female: solid line with circles; Male: dashed line with triangles. Sample sizes per bin (female/male):<40 = 20/10; 40–49 = 23/10; 50–59 = 35/10; 60–70 = 25/4; >70 = 10/5.

As illustrated in **Figure 2**, vBMD declined more steeply in women, especially after age 50, suggesting accelerated postmenopausal bone loss (14). In contrast, vBMD in men remained relatively stable across age groups. Consistently, vertebral cBMD (%) decreased from normal to osteopenia to osteoporosis.

Notably, the mean lumbar DXA T-score in the QCT-normal group was −1.42, within the DXA-defined osteopenia range (15, 16), indicating DXA–QCT discordance. This discrepancy is likely attributable to methodological differences: DXA measures areal BMD that combines cortical and trabecular compartments and is susceptible to artifacts from degenerative changes and calcifications, whereas QCT isolates trabecular vBMD. In PHPT, where cortical bone is preferentially lost, DXA may underestimate bone status earlier than QCT (17, 18). Moreover, younger patients in the QCT-normal group had fewer degenerative changes, reducing artifactual DXA overestimation and further accentuating the discordance. In this subgroup, cBMD (%) of −52.1% to −50.6% suggests subtle trabecular deficits compatible with early metabolic bone loss, potentially accounting for bone pain or low-trauma fractures observed in some patients who have not yet reached QCT-defined osteoporosis thresholds (17, 19).

### 3.2 Correlation between vertebral cBMD and QCT-vBMD

**Figure 3:**
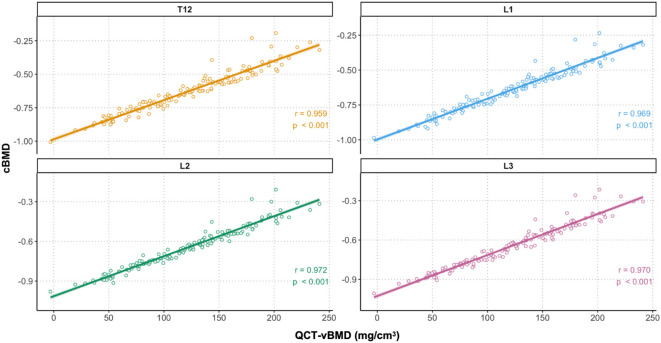
Correlation between vertebral cBMD and QCT-vBMD by level (T12–L3). Scatter plots with linear fits and 95% confidence bands. Pearson correlation coefficients (P< 0.001 for all levels) are shown in each panel.

To evaluate the validity of cBMD in reflecting bone density, we assessed its correlation with QCT-vBMD. Vertebral cBMD showed strong linear correlations with QCT-vBMD across T12–L3 (all r > 0.95, P< 0.001; **Figure 3**) and high inter-vertebral consistency, with r = 0.985–0.994 (**Table 2**; **Supplementary Tables S1**, **S2**). Reliability analysis demonstrated excellent internal consistency (Cronbach’s α = 0.997) and a dominant first component on principal component analysis (explaining 99.2% of variance), supporting the feasibility of simplified single-level assessment. Together, these findings indicate that cBMD closely reflects QCT-vBMD and is robust across vertebral levels in PHPT. As cBMD is expressed as a unitless percentage rather than an absolute volumetric measure, Bland–Altman analysis against QCT-vBMD was considered to have limited interpretability and was therefore not performed.

**Table 2:** Pearson correlations between vertebral cBMD and QCT-vBMD, and inter-vertebral cBMD correlations.

	QCT-vBMD	T12-cBMD	L1-cBMD	L2-cBMD	L3-cBMD
QCT-vBMD	1.000	—	—	—	—
T12-cBMD	0.959	1.000	—	—	—
L1-cBMD	0.969	0.990	1.000	—	—
L2-cBMD	0.972	0.987	0.994	1.000	—
L3-cBMD	0.970	0.985	0.989	0.994	1.000

Pearson correlation coefficients (r); all P< 0.001 (n = 175). QCT-vBMD is reported in mg/cm³; cBMD as a dimensionless percentage deviation from a trabecular reference.

### 3.3 Diagnostic performance of vertebral cBMD for osteoporosis and low bone mass

**Figure 4:**
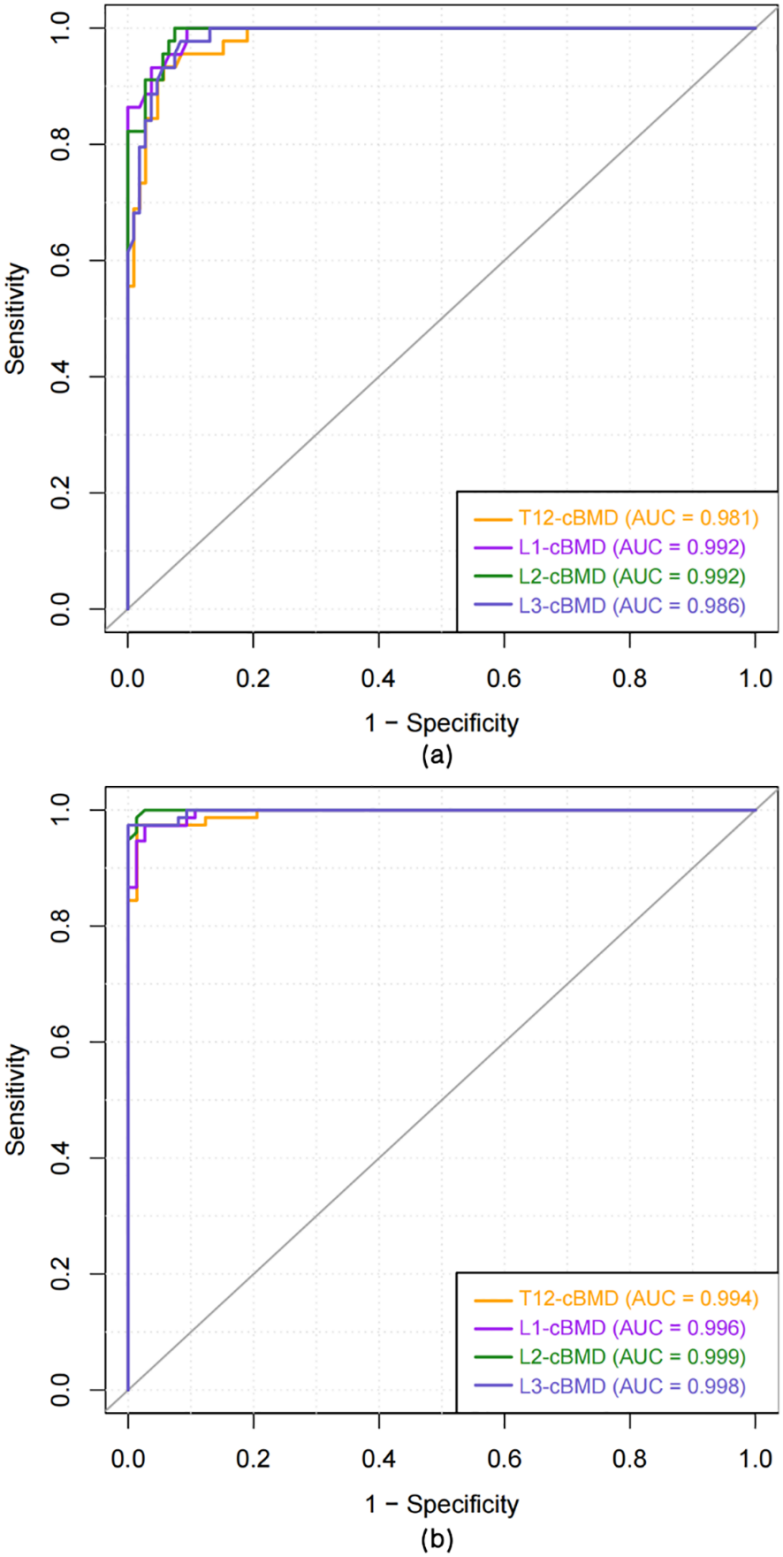
ROC curves for vertebral cBMD by level (T12–L3) diagnosing **(A)** osteoporosis and **(B)** low bone mass. AUC values are indicated. All vertebral levels achieved AUC > 0.98, with L2 performing best. Sensitivity, specificity, optimal cutoffs, and Youden indices are summarized in **Supplementary Table S3**.

ROC analysis showed that vertebral cBMD provided excellent diagnostic performance for both osteoporosis and low bone mass (definitions per Methods) (**Figure 4**; **Table 3**; **Supplementary Table S3**). Across T12–L3, all cBMD levels yielded uniformly high AUCs (>0.98). For osteoporosis detection, L2-cBMD performed best (AUC = 0.992), with 100% sensitivity and 92.5% specificity at the optimal cutoff; L1 showed comparable accuracy (AUC = 0.992). For low bone mass, L2-cBMD reached the highest accuracy (AUC = 0.999, sensitivity and specificity = 98.7%). Overall, vertebral cBMD demonstrated robust diagnostic performance at all levels, with L2 showing marginally superior discrimination. Accordingly, L2-cBMD was entered into the multivariable models as the representative imaging predictor.

**Table 3:** Diagnostic performance of vertebral cBMD for osteoporosis and low bone mass.

Diagnosis	Variable	AUC (95% CI)	Sensitivity	Specificity
Osteoporosis	T12-cBMD	0.981 (0.960–1.000)	93.3%	94.3%
	L1-cBMD	0.992 (0.980–1.000)	100.0%	90.6%
	L2-cBMD	0.992 (0.980–1.000)	100.0%	92.5%
	L3-cBMD	0.986 (0.970–1.000)	97.7%	91.6%
Low bone mass	T12-cBMD	0.994 (0.990–1.000)	97.4%	98.6%
	L1-cBMD	0.996 (0.990–1.000)	97.3%	97.3%
	L2-cBMD	0.999 (1.000–1.000)	98.7%	98.7%
	L3-cBMD	0.998 (0.990–1.000)	97.3%	100.0%

AUC values are shown with 95% confidence intervals; sensitivity and specificity are percentages. Optimal cutoffs and Youden indices are provided in **Supplementary Table S3**.

### 3.4 Incremental value of L2-cBMD in multivariable models for osteoporosis and association with PTH

**Figure 5:**
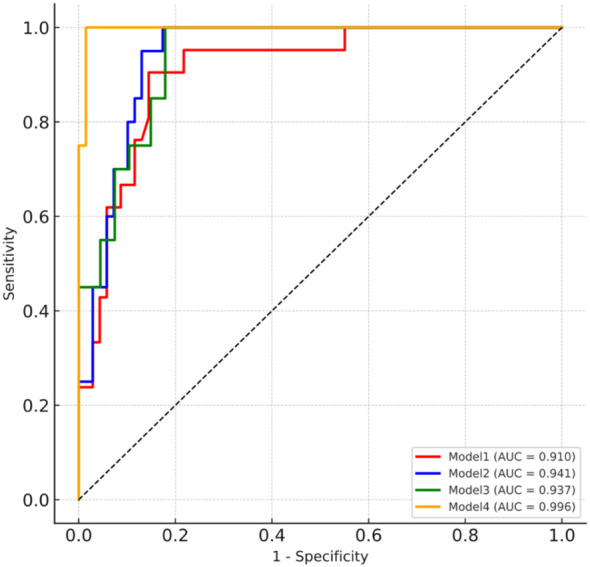
ROC curves of stepwise logistic models for osteoporosis. Model 1: age, sex, BMI; Model 2: Model 1 + serum calcium, PTH, 25(OH)D; Model 3: Model 2 + osteocalcin, PINP, CTX; Model 4: Model 3 + L2-cBMD.

To assess the incremental value of cBMD, stepwise logistic regression models were constructed for QCT-defined osteoporosis. Addition of L2-cBMD (Model 4) significantly improved diagnostic performance compared with Model 3, increasing the AUC from 0.937 to 0.996 (ΔAUC = 0.059; DeLong P< 0.001) (**Figure 5**). Variable importance analysis further identified L2-cBMD as the strongest independent predictor, surpassing traditional predictors such as age, sex, BMI, and bone turnover markers (**Supplementary Table S4**).

Multivariable linear regression analysis demonstrated differential associations between biochemical parameters and imaging-derived bone metrics. Age was the strongest predictor for both QCT-vBMD and cBMD (both P< 0.001). After adjustment for age, sex, BMI, and bone turnover markers, serum PTH levels showed a significant independent negative association with cBMD (β = −0.006, P = 0.010), whereas no significant association was observed between PTH and QCT-vBMD (P = 0.398) (**Table 4**).

**Table 4:** Multivariable linear regression analysis of QCT-vBMD and L2-cBMD in PHPT patients.

Variable	QCT-vBMD (mg/cm³) β	P value	L2-cBMD (%) β	P value
Intercept	230.28	<0.001	-26.14	0.002
Age (years)	-2.70	**<0.001**	-0.86	**<0.001**
PTH (pg/mL)	0.007	0.398	0.006	0.010*
BMI (kg/m²)	1.54	0.185	0.28	0.347
Sex (female)	-7.57	0.400	-3.42	0.144
25(OH)D (ng/mL)	0.06	0.796	0.03	0.611
CTX (ng/mL)	-2.85	0.137	-0.64	0.195
PINP (ng/mL)	-0.002	0.897	-0.003	0.455
BALP (U/L)	-0.11	0.633	-0.02	0.691
Model R²	0.655		0.764	

QCT-vBMD, quantitative computed tomography-volumetric bone mineral density; cBMD, percentage change in bone mineral density; PTH, parathyroid hormone; BMI, body mass index; 25(OH)D, 25-hydroxyvitamin D; CTX, C-terminal telopeptide of type I collagen; PINP, procollagen type I N-terminal propeptide; BALP, bone-specific alkaline phosphatase.

β coefficients represent the change in bone density marker per unit increase in each predictor variable; Bold P values indicate statistical significance (P< 0.05); ^*^Significant association unique to L2-cBMD model.

## 4 Discussion

This study demonstrates that cBMD, a vertebral bone density index derived from routine CT images, is strongly correlated with QCT-vBMD and exhibits excellent inter-vertebral consistency across T12–L3. cBMD achieved uniformly high diagnostic accuracy for both osteoporosis and low bone mass and provided significant incremental value when incorporated into multivariable logistic regression models. Collectively, these findings support cBMD as a robust and reproducible adjunct for opportunistic bone density assessment in patients with PHPT.

Osteoporosis is a major global public health challenge, and early identification of skeletal involvement is critical for fracture prevention and risk stratification (20–23). In recent years, opportunistic use of routine CT scans for bone health assessment has gained increasing attention, enabling extraction of clinically relevant skeletal information without additional radiation exposure or cost. Early studies primarily benchmarked against DXA (24–26), whereas more recent investigations have adopted QCT-vBMD as the reference standard, given its ability to directly quantify trabecular bone. Deep learning–based opportunistic CT approaches have demonstrated high diagnostic performance, with reported AUCs ranging from 0.927 to near-perfect discrimination (27–29). Within this context, the cBMD approach evaluated in the present study achieved comparable diagnostic accuracy (L2-cBMD AUC 0.992 for osteoporosis and 0.999 for low bone mass) while offering a simplified, phantom-less workflow. Importantly, cBMD also provided significant incremental discrimination beyond demographic and biochemical models, underscoring its potential clinical utility.

The clinical relevance of these findings is evident in PHPT, a condition characterized by complex skeletal remodeling and increased fracture risk. Accurate BMD assessment is pivotal for disease evaluation and directly informs the indication for parathyroidectomy (18, 30). While PHPT is classically associated with preferential cortical bone loss (typically assessed at the distal radius), vertebral trabecular bone status remains clinically critical and is routinely used in both DXA and QCT guidelines to direct management. Notably, in our cohort, certain patients classified as having normal bone mass by QCT-vBMD exhibited mild reductions in cBMD. This suggests that subtle trabecular alterations may occur before conventional volumetric thresholds are reached. These observations support the utility of vertebral cBMD as a complementary indicator of skeletal involvement rather than a replacement for established modalities. In clinical practice, for PHPT patients with available CT imaging, a significantly reduced cBMD could serve as a trigger for formal densitometric evaluation or closer monitoring, thereby complementing current DXA-based criteria in guiding therapeutic decisions.

An additional finding of interest was that serum PTH levels showed an independent association with cBMD but not with QCT-vBMD after adjustment for age and other covariates. This difference should be interpreted as an observational association rather than definitive mechanistic evidence. One possible explanation is that the cBMD measurement strategy, which samples a broader trabecular region that extends towards the subcortical compartment, whereas vBMD is estimated predominantly from the central trabecular core. Such difference may allow cBMD to capture subtle PTH-related skeletal alterations that are less evident in central vBMD measurements. Nevertheless, the biological basis and clinical implications of this association remain to be established. Further studies incorporating peripheral skeletal assessments and longitudinal follow-up are required to clarify the biological basis and clinical significance of this finding.

From a methodological perspective, several features may contribute to the robust performance of cBMD. Unlike absolute Hounsfield unit–based metrics, cBMD is expressed as a unitless percentage deviation normalized to a fixed trabecular reference, which may reduce scanner-dependent variability within a single-center setting. Prior studies have shown that QCT-vBMD can be influenced by ROI placement, vertebral level, scanning parameters, and phantom calibration (10, 31, 32). In addition, averaging attenuation across three axial slices per vertebra likely mitigates local heterogeneity and focal artifacts, yielding a more stable estimate of trabecular density. Because cBMD is phantom-independent, it may be particularly suitable for opportunistic and longitudinal assessment in routine CT workflows, although this hypothesis warrants prospective validation.

Notably, L2 emerged as the most representative vertebral level, providing balanced sensitivity and specificity and the highest AUC in our cohort. This finding is consistent with prior opportunistic CT studies that identified L2 or the L1–L3 average as a reliable lumbar site for trabecular bone assessment (24, 33). Importantly, cBMD demonstrated uniformly excellent diagnostic performance across T12–L3, and the very high inter-vertebral correlations highlight its robustness across levels. While L2 offers practical advantages as a single-level predictor, the consistency across vertebrae supports the generalizability of cBMD for both clinical and research applications.

Our study has several limitations. First, this was a retrospective, single-center analysis, which may introduce selection bias and limit generalizability. Second, cBMD relied on a locally derived trabecular reference, and its portability across different CT scanners and acquisition protocols has not been systematically validated. Third, cBMD was derived using manual ROI delineation, and intra- and inter-reader reproducibility were not formally assessed, highlighting the need for future automated segmentation approaches. Additionally, established osteoporosis risk factors (smoking, alcohol use, prior fractures) and disease duration were not systematically available in this retrospective dataset. Peripheral skeletal assessments, such as distal radius DXA or high-resolution peripheral QCT, were also not routinely performed and were unavailable for inclusion, limiting evaluation of cortical compartments that are preferentially affected in PHPT. Finally, the present analysis was cross-sectional and focused on diagnostic performance rather than longitudinal outcomes. Future multicenter, prospective studies incorporating standardized imaging workflows and fracture endpoints are warranted to confirm the broader applicability of cBMD.

## 5 Conclusion

This study validates cBMD, a vertebral bone density index derived from routine CT, as a feasible and accurate tool for opportunistic assessment of osteoporosis in PHPT patients. cBMD showed strong correlation with QCT-vBMD, consistently high diagnostic accuracy across T12–L3, provided incremental discriminative value when incorporated into multivariable models, and demonstrated independent sensitivity to PTH. As a phantom-less, standardized metric obtainable from existing CT data, cBMD may serve as a practical adjunct to conventional bone densitometry for identifying skeletal involvement in PHPT. Further prospective, multicenter studies with automated implementation and fracture outcomes are warranted to define its broader clinical utility.

## Data Availability

The original contributions presented in the study are included in the article/**Supplementary Material**. Further inquiries can be directed to the corresponding author.
