# Supplementary material for: Opportunistic osteoporosis screening in primary hyperparathyroidism using routine CT: validation of a standardized vertebral cBMD index
Source: Front Endocrinol (Lausanne). 2026 Feb 13;17:1710379. doi: 10.3389/fendo.2026.1710379 (PMC12945808; doi:10.3389/fendo.2026.1710379)

Supplementary TABLES

Table S1. QCT-vBMD vs vertebral cBMD: Pearson r with 95% CI and P value.

Pair	r	95% CI	P value
QCT-vBMD vs T12-cBMD	0.959	0.944 to 0.969	<0.001
QCT-vBMD vs L1-cBMD	0.969	0.962 to 0.979	<0.001
QCT-vBMD vs L2-cBMD	0.972	0.961 to 0.978	<0.001
QCT-vBMD vs L3-cBMD	0.970	0.956 to 0.975	<0.001

Table S2. Inter-vertebral cBMD correlations: Pearson r with 95% CI and P value.

Pair	r	95% CI	P value
T12-cBMD vs L1-cBMD	0.990	0.987 to 0.993	<0.001
T12-cBMD vs L2-cBMD	0.987	0.983 to 0.990	<0.001
T12-cBMD vs L3-cBMD	0.985	0.980 to 0.989	<0.001
L1-cBMD vs L2-cBMD	0.994	0.992 to 0.996	<0.001
L1-cBMD vs L3-cBMD	0.989	0.985 to 0.992	<0.001
L2-cBMD vs L3-cBMD	0.994	0.992 to 0.996	<0.001

Summary: average r (QCT-vBMD vs cBMD levels) = 0.968. Average inter-cBMD correlation = 0.990; Cronbach's α (four vertebral levels) = 0.997. The first principal component of the 4×4 cBMD correlation matrix explains 99.2% of the variance.

Notes: Fisher's z for confidence intervals; P values are all <0.001; n = 175 for all calculations.

Table S3. Diagnostic performance details for vertebral cBMD.

Diagnosis	Variable	Optimal cutoff	AUC	95% CI	Sensitivity	Specificity	Youden index
Osteoporosis	T12-cBMD	-0.735	0.981	0.960 – 1.000	93.3%	94.3%	0.876
	L1-cBMD	-0.733	0.992	0.980 – 1.000	100.0%	90.6%	0.906
	L2-cBMD	-0.747	0.992	0.980 – 1.000	100.0%	92.5%	0.925
	L3-cBMD	-0.753	0.986	0.970 – 1.000	97.7%	91.6%	0.893
Low bone mass	T12-cBMD	-0.636	0.994	0.990 – 1.000	97.4%	98.6%	0.960
	L1-cBMD	-0.649	0.996	0.990 – 1.000	97.3%	97.3%	0.946
	L2-cBMD	-0.653	0.999	1.000 – 1.000	98.7%	98.7%	0.974
	L3-cBMD	-0.666	0.998	0.990 – 1.000	97.3%	100.0%	0.973

Notes: QCT-vBMD thresholds: osteoporosis < 80 mg/cm³; low bone mass < 120 mg/cm³. Youden index (J) = sensitivity + specificity − 1. Optimal cutoff refers to the cBMD threshold from ROC analysis. All sensitivity and specificity values are reported as percentages.

Table S4. Variable importance in the final stepwise Model 4 based on absolute standardized coefficients.

Variable	Standardized coefficient (β_std)	|β_std|	Rank
L2-cBMD	-2.554	2.554	1
Sex	-0.523	0.523	2
Age	0.382	0.382	3
CTX	0.266	0.266	4
BMI	-0.238	0.238	5

Notes: Variables shown are those retained in the final stepwise Model 4. Candidate variables entered were Age, Sex, BMI, serum calcium, PTH, 25-OH vitamin D,

osteocalcin, PINP, CTX, and L2-cBMD. Variable importance was ranked by absolute standardized coefficients (|β_std|). Coefficients are from logistic regression with QCT-defined osteoporosis as the outcome.

Supplementary FIGURE

Figure S1. QCT-vBMD measurement.

Semi-automatic ROIs placed at L1 – L3 using Mindways QCT Pro. Volumetric BMD was obtained via asynchronous phantom calibration, and the mean L1 – L3 value was used as the diagnostic index.

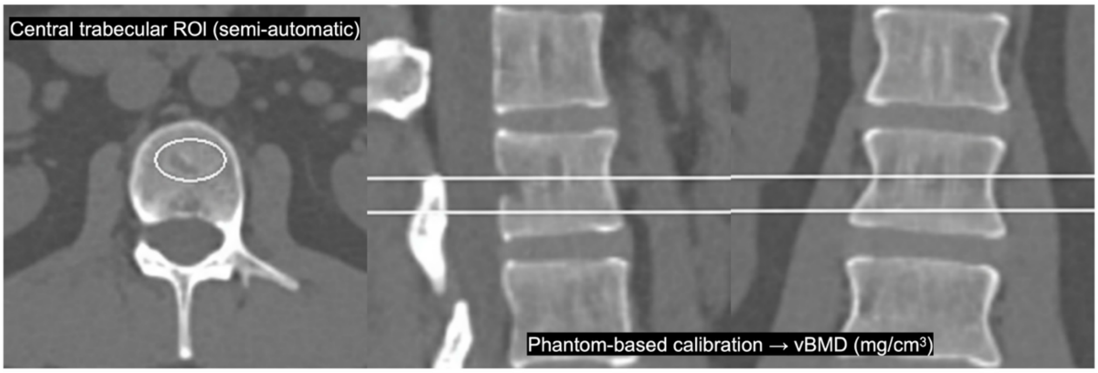

Supplement: Supplementary file 1 [file DataSheet1.pdf]
